# HIV/AIDS, Tuberculosis, and Malaria in Pregnancy

**DOI:** 10.1155/2012/140826

**Published:** 2012-04-22

**Authors:** Oliver Ezechi, Karen Odberg Petterson, Josaphat Byamugisha

**Affiliations:** ^1^Maternal and Reproductive Health Research Unit, Division of Clinical Sciences, Nigerian Institute of Medical Research, 101212 Yaba Lagos, Nigeria; ^2^Division of Global and International Health, Faculty of Medicine, Lund University, 20502 Malmo, Sweden; ^3^Department of Obstetrics and Gynecology, School of Medicine, Makerere University, College of Health Sciences, P.O. Box 7072, Kampala, Uganda

HIV/AIDS, tuberculosis (TB), and malaria (ATM) are 3 major global public health threats that undermine development in low- and middle-income countries [1]. 

Approximately 5 million lives are lost annually as a result of these infections, with substantial humanitarian, economic, and social impact, which is still not fully measured [1]. The three infections are not only associated with poverty but also occur in the same geographic zone and have major public health implications [2]. The consequences of interactions between the diseases are particularly serious for reproductive health. They often intersect in pregnancy, resulting in poor maternal and fetal outcomes. As access to their treatment is increasing in low-income countries and new and cheaper alternative drugs are being deployed, there is also the potential for interactions between their treatments, which affects the efficacy of each drug.

The co-infected pregnant women are at a very high risk of anaemia and infection of the placenta; hence a considerable proportion of children born to women with HIV, TB and malaria infection have low birth weight and are more likely to die during infancy [3–5]. Also, there is the risk of mother to child transmission of the infections.

Globally, an estimated 342,900 maternal deaths occur annually and more than 99% of these occur in low- and middle-income countries [6]. It is barely four years to the target for the attainment of Millennium Development Goal (MDG) 5, which seeks to reduce the maternal mortality ratio (MMR) by three-fourths. The three leading causes of these deaths have remained hemorrhage, infections, and hypertensive disorders [6, 7]. Local and regional variations do exist, however in sub-Saharan Africa, and Asia, infection as a result of HIV epidemic, resurgence of TB, and the ubiquitous malaria is a major cause [7].

Malaria is the most common of the three infections and it is estimated that over 50 million women are exposed to the risk of malaria in pregnancy annually [8]. Malaria infection in pregnancy results in substantial maternal, fetal, and infant morbidity and mortality, accounting for 75 000–200 000 infant deaths yearly [9]. It causes diverse adverse pregnancy outcomes, including maternal anaemia and low birth weight due to preterm delivery and fetal growth restriction. Pregnant women are more susceptible than nonpregnant women to malaria, and this susceptibility is greatest in the first and second pregnancy. Susceptibility to malaria during pregnancy probably represents a combination of immunological and hormonal changes associated with pregnancy, combined with the unique ability of a subset of infected erythrocytes to sequester in the placenta [9].

In the same settings of malaria, HIV infection has continued to remain a major public health challenge and thus have been described as a deadly combination, especially in pregnancy [5]. Available evidence confirms that HIV-positive women are at greater risk of maternal death and morbidity [10]. In high burden areas, which also have the highest rates of maternal mortality, HIV is beginning to reverse the progress in safe motherhood [10]. HIV-infected women confront stigma, discrimination, and violence that compromise their health and may put their lives and their offspring at risk. In some situations they have been refused services for labour and delivery and/or have been given substandard services [10]. The estimates of the contribution of HIV and TB infections to overall maternal mortality vary greatly by region [11]. All are certainly underestimates because the HIV status of many women is not known, and HIV contributes to increased mortality related to pregnancy even beyond 42 days postpartum, the period during which maternal health is calculated [5]. Areas with high HIV prevalence and high maternal mortality also have lower rates of antiretroviral treatment and higher levels of diseases and conditions that can be exacerbated by both HIV infection and pregnancy [12].

There are several ways in which HIV is believed to contribute to elevated maternal mortality and morbidity. HIV infection is associated with an increase in direct complications such as anaemia, postpartum haemorrhage, and puerperal sepsis [11, 13]. In addition, both HIV infection and pregnancy may increase susceptibility to other diseases that affect pregnancy outcome such as tuberculosis, malaria, and pneumonia [9, 12, 14]. Pregnancy itself renders women more vulnerable to acquiring HIV infection. Finally, women who are HIV positive and pregnant may receive lower quality care for both pregnancy and HIV due to discrimination by health care providers [10].

Globally, tuberculosis has increased over the past two decades, with the largest burden of disease borne by impoverished communities in the same geographic areas where malaria and HIV are endemic [13]. While it is known that the deterioration in socioeconomic conditions, poorly functioning national tuberculosis-control programmes, and multidrug resistance have to varying extents contributed to the resurgence of tuberculosis, the main driving force has been the pandemic of HIV-1 infection [13]. A considerable overlap exists in the age groups commonly affected by tuberculosis and HIV [15, 16].

The current understanding of the human immune response to malaria, HIV, and TB leads us to expect the three infections to influence the clinical outcome of each other as well as that of pregnancy. Each of the infections is expected to accelerate the progression of the other and thus impacts heavily on pregnancy, not only in terms of the morbidity and mortality but also on the transmission of the infections to the unborn child [3–5, 8]. The effects of HIV infection on maternal health are superimposed on those of malaria in low-income countries, where over 1 million pregnancies annually are complicated by malaria-HIV coinfection [3, 4]. With the documented 20–30% rate of HIV and TB coinfection in these same settings, it is safe to state that at any given time, more than 300,000 pregnancies are triply infected by HIV, malaria, and TB. Such pregnancies are associated with higher malaria parasite densities, HIV viral load, and a higher risk of maternal anaemia, low birth-weight, and perinatal and infants morbidity and mortality [3–5, 8]. While HIV and TB infection affects all pregnancies, the HIV-associated risk of malaria is consistently greater in multigravida [3–5, 8]. Furthermore there are risks of poorer response to malaria prophylaxis and treatment with HIV and also adverse drug reaction during pregnancy [9].

At the clinical level, the interactions between the infections are complex and bidirectional [9]. Although the morbidity and mortality attributable to each infection in the presence of the other are not fully understood, interactions that are likely to have significant clinical consequences have been described [9, 14]. Each infection exacerbates the other: Malarial and TB infections of the placenta increase placental viral load, thus increasing the risk of vertical transmission of HIV. On the other hand, HIV-infected pregnant women have reduced antimalarial and TB immunity and higher prevalence rates of malaria and TB [15, 16]. TB-infected pregnant women are more susceptible to HIV and malaria infection compared to their noninfected counterparts [3–5].

Though the burden and the consequences of the interaction and intersection of the three infections in pregnancy are frightening, the real tragedy is the failure to make low cost and effective interventions tools available to the women. These tools and interventions include prevention of mother to child transmission of HIV (PMTCT), Intermittent Prophylactic Therapy (IPTp), Long-Lasting Insecticide Treated Nets (LLINs), and infection control practices (ICPs). The prize of the slow or nondeployment of evidence-based and low cost tools is the avoidable human tragedy called maternal and infant mortality.

The regions with high HIV, malaria, and TB burden further suffer from the world's most pronounced crisis in human resources for health [18]. This shortage is compounded by the discussed infections especially the HIV/AIDS pandemic. It is therefore only reasonable to consider human resource issues while trying to prevent and control the infections. One strategy that has been suggested and evaluated is the reassignment of clinical roles by shifting tasks to different cadres of health workers. For examples nurses and midwives may become involved in prescribing drugs, lay counselors involved in testing, and patients may be engaged in taking over some elements of their own care [18].

Halting and reversing the human tragedy due to the deadly trio of HIV, malaria, and TB requires not only the deployment of the evidence-based prevention and management tools already available but also the integration of various services into the existing maternal and child health care services utilizing available human resource. However, the best model for deployment of such tools in low-income countries has not yet been identified. Operational research is therefore required to determine the best models of integration and deployment of these services through testing of existing models

Effort at addressing the challenge of HIV, TB, and malaria in pregnancy should address all sexual and reproductive health services as one entity rather than in units. Many of the interventions that would improve their outcomes such as family planning, antenatal and delivery care, including access to emergency obstetric services are general issues applying to all women. The integration of TB, malaria, and HIV services into existing maternal, newborn, and child health care programmes and task shifting seems to be the ideal strategy to reverse the human toll due to the three infections.

*Oliver Ezechi* *Oliver Ezechi* *Karen Odberg Pettersson* *Karen Odberg Pettersson* *Josaphat Byamugisha* *Josaphat Byamugisha*
